# Treatment of locally advanced and metastatic bladder cancer

**DOI:** 10.4103/0970-1591.38609

**Published:** 2008

**Authors:** Makarand V. Khochikar

**Affiliations:** Department of Uro-Oncology, Siddhi Vinayak Ganapati Cancer Hospital, Miraj, India

**Keywords:** Locally advanced bladder cancer, neoadjuvant chemotherapy, radical cystectomy

## Abstract

**Background:**

There is a significant variation in the treatment strategies adopted for the treatment of locally advanced T3b, T4a, N1-3 and metastatic bladder cancer. There is increasing evidence that we would be able to offer them some benefit in terms of disease-free survival and improving the quality of life. This article is aimed at reviewing the current literature on the treatment strategies in locally advanced and metastatic bladder cancer.

**Materials and Methods:**

Extensive literature search was done on Medline/Pubmed from 1980-2007 using the key words - treatment of locally advanced, metastatic bladder cancer. Standard textbooks on urology, urologic oncology and monograms were reviewed. Guidelines such as National Comprehensive Cancer Network guidelines, European Urology Association guidelines and American Urology Association guidelines were also studied.

**Results and Conclusions:**

There is a place for radical cystectomy in locally advanced T3b-T4 and N1-3 bladder cancer. Radical cystectomy alone rarely cures this subgroup of patients. There is increasing evidence that meticulous surgical clearance and extended lymphadenectomy has significant impact on disease-free survival. Adjuvant chemotherapy has been found to be effective in terms of recurrence-free survival and better than cystectomy alone. Neoadjuvant chemotherapy followed by radical cystectomy also has beneficial effects in terms of downstaging the disease and improving recurrence-free survival. This perioperative chemotherapy (adjuvant/neoadjuvant) has 5-7% survival benefit and 10% reduction in the death due to cancer disease. Excellent five-year survival rates have been achieved in patients achieving pT0 stage at surgery following chemotherapy (around 80%) and overall 40% five-year survival in node positive patients, which is promising. Though practiced widely, perioperative chemotherapy is not considered as a standard of care as yet. Current ongoing trials are likely to help us in reaching a consensus over this. There is no role of preoperative or postoperative radiotherapy in locally advanced/metastatic bladder cancer except in non TCC bilharzial/squamous cell carcinoma of bladder. Use of nomograms and prognostic factor evaluation may help us in the future in predicting the disease relapse and may help us in tailoring the treatment accordingly. Newer and more effective chemotherapeutic drugs and ongoing trials will have a significant impact on the treatment strategies and outcome of these patients in the future.

Bladder cancer is the fourth most common cancer amongst all the cancers in men and ranks 11^th^ amongst the cancer in women. It has shown almost 20-fold international variation in incidence.[1] The incidence is 40 per 100,000 population in non-Latino whites in the United States and most Western Europeans while in Asians (including Chinese, Japanese and Indians) the incidence is 3-7 per 100,000.[1] In India bladder cancer is the commonest urological cancer.[2] It tops the urological cancer workload in major centers that undertake urological oncology work in India (personal communication).

Treatment of noninvasive (Ta, T1 No Mo- AJCC classification 1992)) bladder cancer is now almost universally the same with minor variation in the type/duration of intravesical therapy when required. Similarly, the treatment of muscle invasive disease stage T2, No Mo is also universally same i.e. Radical Cystoprostatectomy (in men), radical cystectomy ± hysterectomy (in women) with bilateral pelvic LN dissection. (NCCN, EAU, guidelines).[3,4] However, significant variation in the treatment strategies is seen in the treatment of locally advanced - T3b, T4a and N1-N3 disease. Treatment of metastatic bladder cancer (extra pelvic nodal, visceral and distant metstasis) is also a difficult task. A study using the Surveillance, Epidemiology and End Results (SEER) Medicare Database by Deborah Schrag *et al.*,[5] found a marked heterogenicity in the strategies used to treat muscle invasive bladder cancer [Table 1a,b]. The study included 4664 patients with muscle invasive bladder cancer with age 65 years or older. The variation in the treatment was attributed to the lack of informative clinical trials, presence of co-morbid illness, patient or physical preferences or access to care.

**Table 1a:** Treatment patterns among Stage III bladder cancer within six months of diagnosis (N = 1096)[5]

Surgery only	42%
Chemotherapy + surgery	07%
Chemotherapy only	06%
Chemotherapy + radiation	08%
Radiation only	11%
Radiation + surgery	04%
None	18%
All three modalities	04%

**Table 1b:** Treatment patterns among Stage IV bladder cancer within six months of diagnosis (N = 1577)[5]

Surgery only	21%
Chemotherapy + surgery	10%
Chemotherapy + radiation	12%
Radiation only	11%
Chemotherapy only	12%
Radiation + surgery	02%
None	26%
All three modalities	05%

This article is aimed at reviewing the current treatment strategies, the logistics behind them, results of various trials/studies for locally advanced - T3b, T4a, N1-N3 disease and metastatic bladder cancer.

The major issues that are discussed here in the treatment of locally advanced bladder cancer are:

- Is there a role of radical cystectomy in locally advanced/metastatic bladder cancer?
- Surgery alone or adjuvant chemotherapy and its effectiveness?
- What is the role of neoadjuvant chemotherapy followed by surgery?
- Neoadjuvant chemotherapy versus adjuvant chemotherapy - which one is better?
- Role of radiotherapy - pre and post cystectomy in locally advanced bladder cancer.
- Role of post-chemo surgery in unresectable and metastatic bladder cancer.
- Use of nomograms, prognostic factors and future perspectives.

## ROLE OF RADICAL CYSTECTOMY IN T3b, T4a DISEASE - IS IT WORTH DOING?

Radical cystectomy with curative intent for Stage T1-T2 and node negative patients has approximately 50-60% five-year survival rate.[7] The survival rate drops significantly with increasing stage and more importantly with nodal involvement. This is in the range of 26-44% in pT3b-4 No patients and 13-29% in N2-N3 patients [Table 2].[6–11]

**Table 2:** Survival after radical cystectomy alone for pT3b - T4

Study, year (ref)	No. of patients	Five-year survival
Dalbagni, 2001[6]	129	26%
Stein, 2001[7]	254	44%
Maderbacher, 2003[8]	111	38%
Herr, 2003[9]	353	42%

Radical cystectomy is a supra-major operation, has a perioperative morbidity of around 5-30% and mortality of less than 5%.[12] There are more sensitive issues like body image, negative psychological impact and recurrent cost involved, in patients who undergo urinary diversion with radical cystectomy. In the background of these issues and dismal outcome and low survival rates, one would really question the usefulness of radical cystectomy for locally advanced and metastatic bladder cancer.

However, the study of various trials and meta-analysis on radical cystectomy in locally advanced bladder cancer favorably argues towards its usefulness. Its usefulness is not just in the palliation of symptoms, but there is a significant recurrence-free survival in these patients. The key factor that is important in delivering the best out of radical cystectomy is how well one performs this radical surgery. A good and meticulous clearance of the pelvis along with nodal clearance is extremely important in achieving better survival rates. There has been a lot of emphasis on extended lymphadenectomy[13] that enables the surgeon to clear the nodal disease as much as possible and also gives a correct pathological staging. This pathological staging forms the basis of prognostification and planning adjuvant treatment in these patients. Many studies show that extended lymphadenectomy improves disease-free survival.[13–15] There is no randomized trial on standard versus extended lymphadenectomy till date, but the results of extended lymphadenectomy underline the importance of how a good and meticulous clearance translates into a better survival.

There has been a lot of emphasis on who should undertake these kind of cystectomies and it has been proved that the ‘high volume’ centers should take up this type of surgery rather than those who do low volume (less than 12-15 a year) and those who are ‘occasional cystectomy’ surgeons.[13]

After all what else can you offer to these patients? Survival outcomes are poor without therapy.[16] Systemic chemotherapy though effective in significant downstaging of the disease and almost complete response in some patients, the relapse rate (local and distant) is very high if not augmented with surgery in locally advanced bladder cancer. Similarly, surgery alone is curative in a very small subset of these patients and adjuvant chemotherapy in this setting has been found to be useful. These statements/views are argued in detail in this article in subsequent sections.

Therefore, it appears that there is a place for radical cystectomy and it is worth doing it in locally advanced bladder cancer.

However, Stage T4b disease i.e. the disease fixed to the pelvic wall and abdominal wall is rarely cured with radical surgery even when used as an adjunct to neoadjuvant chemotherapy. Radical cystectomy is therefore not considered for these patients even when they have severe local symptoms. Palliation in terms of supportive care, radiation for pain relief and hematuria and systemic chemotherapy is considered instead.

## IS RADICAL CYSTECTOMY ADVISABLE IN NODE POSITIVE PATIENTS ?

Involvement of nodes is the most important prognostic factor in invasive bladder cancer. The five-year survival in N1-N3 patients in contemporary series is from 13-29% [Table 3].[6–11]

**Table 3:** Survival after radical cystectomy alone for N2-3 patients

Study, year (ref)	No. of patients	Five-year survival rate
Dalbagni, 2001[6]	39	13%
Stein, 2001[7]	86	24%
Zincke, 2002[10]	24	15%
Mills, 2002[11]	60	29%
Maderbacher, 2003[8]	44	26%
Herr, 2003[9]	108	28%

We often face this situation in any of the following scenarios:

- Node involvement is highly suspected on preoperative CT, MRI and other investigations.
- Preoperative evaluation – no nodes, but on exploration it may be highly suggestive of nodal involvement.
- Grossly involved nodes on preoperative and or intra-operative assessment.

In the first scenario, when the nodal involvement on imaging is doubtful or possibly going to be positive, it is advisable to go ahead and perform radical cystectomy and complete nodal clearance. It is of extreme importance that no gross residual disease is left behind, the clearance is meticulous and one practices extended lymphadenectomy (Level I, II and III) in order to achieve better long-term results. The Level I dissection is limited to the obturator fossa, Level II dissection extends over to the genitor-femoral nerve and typically up to the common iliac bifurcation and Level III dissection includes the pre-sacral tissue and extends up to the origin of the inferior mesenteric artery. This approach gives not only excellent chance of clearance of the disease, but more importantly enables us to have accurate pathological staging. Node negative patients can be observed while node positive patients could be offered adjuvant chemotherapy. If it appears that gross residual disease is going to be left behind it is advisable to close and have neoadjuvant chemotherapy and perform post chemo salvage surgery.

In the second scenario, the same principle applies as discussed above and one should aim at complete clearance in the pelvis and offer chemotherapy in node positive patients.

Decision-making in a scenario where there is gross involvement of pelvic nodes on preoperative and or intra-operative assessment is difficult. This issue was addressed by Herr *et al.*,[17] in a study where a total of 84 patients with grossly node positive (N2-3) status underwent radical cystectomy and extended lymphadenectomy. Twenty patients (24%) survived at 10 years with surgery alone. The overall median survival was 19 months for all patients. In the editorial comment on this article, Michael S Cookson has cautioned the urological community about the fact that these results have been achieved at a center where 800 cystectomies had been performed over a 10-year period, underlying the fact that high-volume centers can achieve these kind of results rather than ‘occasional cystectomy’ centers.

However, the same group had shown less than 20% five-year survival rate in pT3-T4 N+ patients in their earlier experience.[18] The University of Southern California (USC) group has shown 27% 10-year survival rate in 84 patients with five or more positive nodes.[7] It was also an interesting finding by Herr *et al.*, that patients with positive nodes who are more likely to benefit from surgery have primary tumors that are pathologically confined (stage P0-P2) to the bladder rather than extravesical (pT3-4) disease.[18]

In conclusion, it appears that surgery alone offers cure in around 20-25% bulky nodal disease. However, extended lymphadenectomy and systemic chemotherapy has resulted in cure in more than 40% patients in many studies and meta-analysis of adjuvant chemotherapy. Therefore it can be argued that there is a place for radical cystectomy in node positive patients with the advent of use of chemotherapy in perioperative setting.

One has to also remember the potential complications and technical difficulties such as vascular, nerve injuries, and excessive bleeding from the pelvic vessels in dealing with bulky lymph node disease. This again underlines the importance of undertaking these surgeries by high-volume centers to achieve complete clearance and good results.

Surgery would not be curable for patients who have nodal involvement above the inferior mesenteric vessels (above Level III), systemic chemotherapy may be an answer for those.

## RADICAL CYSTECTOMY ALONE VS. ADJUVANT CHEMOTHERAPY

Radical cystectomy alone rarely cures locally advanced bladder cancer.[16] Various trials and meta-analysis have shown the superiority of adjuvant chemotherapy over cystectomy alone. There has been modest to statistically borderline benefit in favor of chemotherapy.

In a non-randomized trial Logothetis *et al.*, compared a group of 71 post cystectomy patients (resected nodal disease, extravesical extension, lympho-vascular invasion or pelvic visceral invasion) whom they had administered CISCA (Cisplatin, cyclophosphamide, doxorubicin) to 62 high-risk and 206 low-risk patients who did not receive chemotherapy. They found that CISCA conferred a two-year disease-free survival advantage to patients who received chemotherapy (70% *vs.* 30%, *P* = 0.00012).[19]

One of the first randomized control trials of adjuvant chemotherapy *vs.* cystectomy alone was carried out at the University of Southern California (USC). Ninety-one patients with pT3-4, N+ were randomized to four cycles of cyclophosphamide, adriamycin (doxorubicin), cisplatin (CAP) or observation. Chemotherapy resulted in a significant improvement in the risk of disease recurrence at three years (0.30 *vs.* 0.54; *P* = 0.011) and in the overall risk of death (0.34 *vs.* 0.50; *P* = 0.099). The median survival of patients on chemotherapy was found to be 4.25 years *vs.* 2.4 years in the observation group. This study was criticized for the methodology of its statistical analysis, fewer patients completing the full course of chemotherapy and sample size etc. However, this was a stimulating study suggesting the potential benefit of adjuvant chemotherapy and practical difficulties in conducting such trials.[20]

Studer *et al.*, in 1994, published their results of a randomized trial of adjuvant chemotherapy with three cycles of cisplatin alone in 77 patients. This did not result in any survival benefit in comparison to observation alone. These results could well be due to the use of a single chemotherapy agent, small sample size and only 65% patients in the treatment arm completing the therapy.[21] There were two more trials-one from Italy[22] and another from Germany[23] showing no significant survival benefit. Despite the limitations and criticism of these three trials, it is still debatable whether neoadjuvant or adjuvant chemotherapy is the preferred mode of therapy.

Knowing the superiority of M-VAC over single agent cisplatin in a metastatic setting[24] studies were undertaken with M-VAC or M-VEC (methotrexate, vinblastine, epirubicin, cisplatin). Stockle *et al.*,[25] randomized 49 patients to observation (23 patients) *vs.* adjuvant three cycles of M-VAC or M-VEC (26 patients). The authors were planning to accrue 100 patients, but the interim analysis was suggestive of the beneficial effects of chemotherapy in the chemotherapy group (*P* = 0.0015), therefore it was prematurely closed. The trial was criticized for only 62% patients in the chemotherapy group completing chemotherapy, patients in the observation group not being offered chemotherapy on relapse and premature closure. The same group subsequently looked at additional 38 patients who had received M-VAC/M-VEC therapy and reviewed the results of 83 patients (49 patients of the trial that was closed +38 patients) and concluded a significant survival benefit in the chemotherapy group on long-term follow-up (38-78 months, *P* = 0.0005).[26] Recently, the same group has now come out with a 10-year data of the same trial suggesting better progression-free survival (13% *vs.* 43.7%), overall survival (17.4% *vs.* 26.9%) and tumor-specific survival (17.4% *vs.* 41.7%).[27]

A prospective randomized trial of M-VAC *vs.* observation was conducted by a Stanford University group. With a median follow-up of 62 months, a significant difference in progression-free survival was found in the chemotherapy group (37 months *vs.* 12 months *P* = 0.01), however, no significant difference in overall survival was noted. This was also closed prematurely noting the usefulness of chemotherapy in interim analysis and offered deferred chemotherapy on progression in the observation group.[28]

Criticisms of the trials favoring adjuvant systemic chemotherapy in advanced bladder cancer have been summarized recently by Sylvester and Sternberg in an article ‘what we do not know and why?’.[29]

The various issues such as small sample size, lack of power to come to conclusions, dropout rates, inability to complete the full course of chemotherapy due to side-effect profile, early closure of the trials have to be kept in the mind while looking at these trials.

A meta-analysis including data from 491 individual patients who have been studied in adjuvant chemotherapy trials was carried out by Advanced Bladder Cancer Meta-analysis Collaboration (ABC).[30] This review also underlines the absolute survival benefit in the chemotherapy group.

Toxicity profile has not been highlighted in detail in many trials of adjuvant chemotherapy. Looking at the incidence of myeolsuppression, sepsis and other toxicities a recent trial was conducted in Germany on patients from 40 uro-oncological centers. A total of 327 patients were studied. The study was aimed at looking into the omission of vinblastin and epirubicin from the M-VEC regime. The trial was cisplatin and methotrexate *vs.* M-VEC, it showed similar five-year recurrence-free survival - 46.3% for cis + metho *vs.* 48.8% for M-VEC. It is noteworthy from this study that five-year recurrence-free survival for node positive patients was >40% in both the arms.[31]

## NEWER AGENTS AND ONGOING TRIALS IN ADJUVANT SETTING

In the light of the success of taxanes and gemcitabine in metastatic bladder cancer, efforts have been made to use them with cisplatin in adjuvant setting. The combination of gemcitabine and cisplatin (GC) has similar survival outcomes with less toxicity compared to M-VAC in advanced or metastatic setting.[32]

There are many ongoing trials looking at adjuvant chemotherapy in locally advanced bladder cancer such as Cancer and Leukemia Group B 90104 (AG-TP- {doxorubicin, gemcitabine then paclitaxel, cisplatin} *vs.* GC), EORTC 30994 (postoperative *vs.* delayed chemotherapy M-VAC or GC), SWOG (MVAC *vs.* no chemotherapy). The results would be interesting and would perhaps help in deciding if this could be a standard of care.

## PRACTICAL ISSUES SUCH AS AGE, PERFORMANCE STATUS AND ALTERED RENAL FUNCTION IN ADJUVANT CHEMOTHERAPY

Management of invasive bladder cancer in elderly patients is always challenging. There has been enough evidence now that radical surgery is safe even in octogenarians.[33] Similarly, chemotherapy in patients age 65 or more has also been studied and has shown no additional morbidity. The important parameter to consider is the performance status.

In a study by Bajorin *et al.*,[34] the presence of baseline Karnofsky performance status (KPS) less than 80% or visceral metastases has a negative impact on overall survival. They analyzed a group of 203 patients with advanced bladder cancer treated with M-VAC. The patients who had visceral metastasis and KPS less than 80% had 0% five-year survival (median survival 9.3 months), while patients who did not have these risk factors had 33% five-year survival (median survival 9.3 months)

Renal insufficiency either due to obstruction or parenchymal disease poses a significant challenge for chemotherapy. Generally, chemotherapy is not advisable when the GFR is below 60 ml/min. There is a general perception that de-obstructing measures such as stents, nephrostomies should be undertaken to normalize the renal function before contemplating chemotherapy. However, one has to be aware that these are the potential sources of infection and could lead to sepsis and make the matters worse during chemotherapy.

Patients with higher creatinine - >2 mg% have significant morbidity; therefore either chemotherapy is not advised in these patients or use of newer combination like GC can be used. Substitution of drugs not dependent on renal function can be another option, such as use of carboplatin, but direct comparison of this drug with cisplatin suggests that carboplatin is inferior in this disease. Many oncologists would be reluctant to make modifications that may abrogate the effectiveness of the therapy. Fractionation of cisplatin dose is another alternative to reduce the nephrotoxicity. However, no comparison with un-fractionated regimes has been made to confirm that there is no loss of efficacy.

Similarly, patients who have ejection fraction < 45% are not considered for M-VAC therapy due to doxorubicin-induced cardiac toxicity.

## NEOADJUVANT CHEMOTHERAPY FOR LOCALLY ADVANCED AND METASTATIC BLADDER CANCER

In the light of excellent response to cisplatin-based chemotherapy in metastatic bladder cancer, many trials have been conducted and are ongoing, studying the role of neoadjuvant chemotherapy followed by cystectomy in locally advanced bladder cancer. The results of the three major trials conducted so far have shown significant downstaging of the disease allowing a better surgical clearance, higher recurrence-free survival, almost 80-90% in pT0 on post cystectomy patients and a small but demonstrable 5-6% absolute survival benefit and 10-13% reduction in risk of death from bladder cancer.

The MRC/EORTC trial used CMV as a regime for neoadjuvant therapy in advanced bladder cancer and found an absolute improvement in survival of 5.5% (*P* = 0.075).[35]

Nordic trials I[36] and II[37] which used neoadjuvant chemotherapy cisplatin/doxorubicin and cisplatin/methotrexate regimes in locally advanced bladder cancer patients have found overall five-year survival of 56% in the neoadjuvant group versus 48% with cystectomy alone, favoring chemotherapy (*P* = 0.04).

A co-operative group randomized study (Southwest Oncology Group - SWOG 8710 - Intergroup - 0080) involving 307 patients with muscle invasive bladder cancer (Stage cT2-T4a) found a significant survival benefit for the neoadjuvant group over the group treated with cystectomy alone. With more than eight years' follow-up the median survival time was 46 months in the cystectomy group versus 77 months in the combination group (*P* = 04). At five years 57% patients in the chemotherapy + surgery group were alive as compared to 43% in the cystectomy alone group. There was 14% reduction in absolute mortality and a 5% improvement in five-year survival rate. Patients who had pT0 at surgery after chemotherapy had 85% five-year survival rate. Further analysis in the SWOG trial has shown that the patients with T3-T4 disease had median survival of 65 months (92 patients treated with M-VAC + cystectomy) versus 24 months (93 patients treated with cystectomy alone). There was 10% reduction in mortality in the combination therapy group in comparison to cystectomy alone and a 20% improvement in five-year survival for T3b-4 tumors.[38]

A study from M. D. Anderson Cancer Center has also addressed the issue of the use of chemotherapy in combination with surgery for locally advanced bladder cancer. A total of 140 patients were randomly assigned to receive either two courses of neoadjuvant M-VAC followed by cystectomy plus three additional cycles of chemotherapy or, alternatively, to have initial cystectomy followed by five cycles of adjuvant chemotherapy. Although there was no difference in outcome between the two groups, 81 patients (58%) remained disease-free with a median follow-up of 6.8 years. In this study, nearly 40% cure rate was achieved in lymph node positive patients. This is a noteworthy finding.[39]

Meta-analysis of all these trials in more than 3000 patients confirms that neoadjuvant chemotherapy improves survival in locally advanced bladder cancer. Platinum-based combination chemotherapy has a 5% absolute survival at five years and a 13% reduction in the risk of death from bladder cancer.[40]

## ADJUVANT/NEOADJUVANT CHEMOTHERAPY FOR T3, T4, N1-3 + DISEASE - WHICH REGIME?

Since 1976, when cisplatin entered clinical trials in bladder cancer as a single agent, chemotherapy in bladder cancer has evolved a great deal. Sequential trials showed its superiority over supportive care and then the era of M-VAC (Methotrexate, vinblastin, doxorubicin and cisplatin) started. Combination chemotherapy such as M-VAC was found to be superior to cisplatin alone[24] and to CISCA (cyclophosphamide, doxorubicin and cisplatin).[41] Since then M-VAC or M-VEC has been studied in many trials as mentioned above. Looking at the toxicity profile, attempts have been made to either eliminate epirubicin and vinblastin from M-VEC (German study) or use gemcitabine + cisplatin combination (GC). Both these regimes have shown equal results with less toxicity; perhaps cost would be an issue to GC.

## NEOADJUVANT CHEMOTHERAPY OR ADJUVANT CHEMOTHERAPY - WHICH ONE TO CHOOSE?

The effectiveness of adjuvant and neoadjuvant chemotherapy has been proved in various studies as discussed above in this article. However, should one proceed with cystectomy followed by adjuvant chemotherapy or neoadjuvant chemotherapy followed by cystectomy in locally advanced cancer?

Each one has its advantages and disadvantages. Cystectomy first approach allows accurate pathological staging, leaves minimal tumor burden if any and adjuvant chemotherapy in turn would be more effective. The criticism for surgery first approach would be delay in the systemic chemotherapy keeping the morbidity of a supra-major surgery in mind and sometimes having not been able to completely clear the disease from the pelvis that perhaps would have been possible with preoperative chemotherapy.

Neoadjuvant chemotherapy followed by cystectomy has the distinct advantage of downstaging the disease and in some cases even the non-resectable lesions have become resectable and even have achieved pT0 stage. The long-term survival in patients having reached pT0 stage has been in the range of >80%. The downside of this approach is delay in surgery due to potential complications of chemotherapy and some patients may not reach the surgery at all. Cystectomy following neoadjuvant chemotherapy has a major impact on the disease-free survival.

A single study has compared these two approaches and has found the outcomes to be very similar.[42]

Use of both preoperative and postoperative chemotherapy was compared with cystectomy and adjuvant chemotherapy in a study by Millikan *et al.*[39] and there was no difference in the efficacy and disease-free survival.

In our experience, we tend to perform surgery first in bulky but completely resectable disease followed by chemotherapy. In case of a large nodal disease, possible invasion of adjacent viscera on preoperative evaluation, we offer neoadjuvant chemotherapy followed by surgery. The patients are fully informed about these two approaches prior to treatment strategy.

## SYSTEMIC CHEMOTHERAPY ALONE ?

Bladder remains at a risk of new tumors despite good clinical response, therefore the studies suggesting partial cystectomy or observation alone or transurethral bladder tumour resections (TURBTs) for small residual disease have been disappointing. Salvage cystectomy remains a preferable option in these cases. Cystoscopy, biopsy, imaging are poor substitutes for surgical resection. It has been found that the so-called complete responders on biopsy or imaging following chemotherapy die of disease relapse if left untreated with surgery.

## POST CHEMO SURGERY FOR UNRESECTABLE AND NODE POSITIVE BLADDER CANCER

Two major studies, one from Herr *et al.*[43] and another from the M. D. Anderson Cancer Center[44] have reported long-term survival in a very select group of patients. These were the patients who had shown significant downstaging of the disease (complete responders). These studies also suggested that a surgical resection of prechemotherapy sites of loco regional disease might improve relapse-free survival.

## POST CHEMO SURGERY FOR METASTATIC BLADDER CANCER

The results of post chemotherapy surgery that clears the pelvis and regional LNs are encouraging, but the same cannot be held true for patients who have undergone metastatectomy of the lesions outside the pelvis. The results of the three reported series (MSKCC, Stanford and MDACC) have shown median survival time of around 30 months with approximately one-third of these patients surviving for three to five years.

## IS SURGERY ADVISABLE IN POOR/MINIMAL RESPONDERS AND NO RESPONDERS TO CHEMOTHERAPY?

One of the important prognostic factors that determines the success of combination therapy (Chemo + surgery) is the degree of response to chemotherapy. As discussed in this article before, the patients who achieve better survival rate are the ones who had major/complete response to chemotherapy and those having non-visceral metastasis. No patient who achieved less than a major response to chemotherapy survived five years in any of the series reported in this article, despite post chemotherapy surgery. Therefore one has to be extremely careful in advising surgery to these patients.

Surgical resection in chemo refractory patients has been rarely rewarding and is rarely reported except in palliation of selected cases. A study from Germany looked at these patients who underwent surgery despite poor response to chemotherapy and found very dismal results.[45]

## ROLE OF PREOPERATIVE RADIATION PRIOR TO CYSTECTOMY IN LOCALLY ADVANCED BLADDER CANCER

The proposition of preoperative radiation seemed to be attractive in the late 80s and early 90s. In fact, this was a routine practice in many oncological centers across the globe. The proponents of this strategy believed in radiotherapy taking care of micro-metastasis and downstaging the primary tumor. However, the results of various trials and studies have now proved that it does not improve the survival.

Six randomized controlled trials have been conducted (four for TCC bladder, two for bilharzial cancer) to address the issue of preoperative radiation with cystectomy vs. cystectomy alone. The four trials on TCC bladder were by Blackard *et al.*[55] in 1972, Slack *et al.*[56] in 1977, Anderstrom *et al.*[57] in 1983, Smith *et al.*[46] in 1997 (SWOG study).

The SWOG study on this issue was initiated in 1982 and the results were published by Smith *et al.*, in 1997. In the SWOG study patients with advanced bladder cancer were treated either with cystectomy alone or with preoperative radiation followed by cystectomy; a total of 124 patients were randomized to preoperative irradiation with 2000 cGy plus radical cystectomy versus radical cystectomy alone. The five-year survival probability was 53% and 43% respectively (*P* = 23). This study demonstrated no significant benefit of preoperative radiation in terms of survival.[46]

Further meta-analysis of these six trials has demonstrated a corrected odds ratio of 0.94 (95% confidence interval, 0.57-1.55), suggesting no benefit from preoperative radiation.[47]

However, the two trials for bilharzial cancer by Awwad *et al.*[58] and Ghoneim *et al.*,[59] have shown survival benefit for locally advanced bilharzial cancer (T3, T4). They have found reduced pelvic recurrence in these patients. The radiation was used in the dose of 4000 cGy.

Non-bilharzial squamous cell carcinoma is an aggressive disease, it has poor prognosis in comparison to TCCC stage to stage. Usefulness of preoperative radiation in these patients was studied by the M. D. Anderson Cancer Centre with the use of 5000cGy radiation in preoperative sitting. This was a retrospective study and they found downstaging in 40% of the patients and improved disease-specific survival.[48]

We do not have any effective adjuvant chemotherapy for squamous cell carcinoma of the bladder, so preoperative radiation appears to be an attractive proposition in bilharzial and non-bilharzial squamous cell carcinoma bladder. Further work is required, especially in our country as we do see many patients with stone disease and recurrent infections succumbing to aggressive squamous cell carcinoma bladder where results are poor.

## ROLE OF POSTOPERATIVE RADIATION FOLLOWING CYSTECTOMY

Radiation to the pelvis following radical cystectomy for locally advanced and node positive patients have very few buyers. Like preoperative radiation, its effectiveness has been questioned in many studies and is not a standard of care. In the light of effective chemotherapy postoperative radiation does not offer any advantage. In fact it adds more complications mainly related to the gastrointestinal tract, wound healing and iatrogenic complications.

However, the studies looking at its role in bilharzial and non-bilharzial squamous cell carcinoma of the bladder are suggesting some usefulness in local control.[49]

## TYPE OF DIVERSION IN LOCALLY ADVANCED BLADDER CANCER

There has not been enough literature suggesting the pros and cons of what could be ideal urinary diversion in case of advanced bladder cancer after radical cystectomy. There was a suggestion that one should not deny neobladders in these patients on the background of lower survival rates, as the quality of life is also important in these patients. However, there are not many studies that have evaluated this issue in the available literature. In the light of inherent problems of various neobladders, possibility of pelvic recurrence, one would not like to add to more morbidity. We are of the opinion that a gold standard ileal conduit or Mainz II pouch works as good as anything in these patients.

It has to be remembered that there is no place for only urinary diversion in case of advanced bladder cancer.

## PROGNOSTIC FACTORS IN DECIDING THE TREATMENT STRATEGIES IN LOCALLY ADVANCED AND METASTATIC BLADDER CANCER

Good performance status, non-visceral metastasis, low-volume lymph node disease, low p-stage (pT1-T2) and no residual disease in the cystectomy specimen (pT0) have been found to be significant prognostic factors predicting recurrence-free survival.[17,34,38] However, would there be a test/tests preoperatively which could help us in decision-making regarding treatment strategies and pretreatment counseling? Work on p53 status of the tumor and use of nomograms are probably inching towards this lately.

Study of p53 status of the tumor in predicting the survival in patients undergoing neoadjuvant chemotherapy was studied at MSKCC.[50] It was found that patients who had wild type p-53 had significantly better survival (77% five-year survival) than who had mutant p53. Further studies are underway by the same group to see if p53 can be used as a biomarker and plan less aggressive surgical treatment in good responders.

## USE OF NOMOGRAMS IN LOCALLY ADVANCED BLADDER CANCER

Currently, treatment strategies for advanced bladder cancer in many oncological centers are based on the data discussed above. Many centers opt for radical cystectomy followed by adjuvant chemotherapy or neoadjuvant chemotherapy followed by surgery alone or additional courses of chemotherapy following surgery. There are no set guidelines for this as yet. The current literature does give us a rough idea about chances of recurrence-free survival, but better ways are now explored with the help of nomograms.

Recently, attempts have been made to use the nomograms in predicting recurrence-free survival.[51,52] The study from the International Bladder Cancer Nomogram Consortium by Bochner *et al.*, had studied 9000 patients from 12 different centers who had undergone radical cystectomy and bilateral pelvic LN dissection for the possible risk of recurrence. The nomograms fared much better than standard AJJC staging and standard pathological subgrouping. Concordance index of nomograms was 0.75 as compared to concordance index of standard TNM AJCC classification (0.68*P* < 0.001) and that of standard pathological subgrouping (0.62*P* = 0.001). The authors have suggested using the nomograms rather than TNM/pathological subgroupings in predicting the recurrence. The same authors have consolidated this data once again recently.[53] Another study by Karakiewicz *et al.*, who had studied 728 patients of advanced bladder cancer has shown that the nomograms were 3.2% more accurate than TNM AJCC classification.[54]

Karakiewicz *et al.* have also recently published their work on the use of precystectomy nomograms for prediction of advanced bladder cancer stage. Unlike post cystectomy nomograms, they found these nomograms not particularly accurate but better than TURBT stage prediction.[54]

These nomograms would certainly be useful in identifying the high-risk and low-risk individuals. The high-risk individuals who are likely to relapse may be offered additional therapy while the low-risk individuals could be observed. These nomograms are not in regular practice everywhere, but would be a future tool in deciding the treatment plan in locally advanced bladder cancer.

## TREATMENT GUIDELINES AT OUR INSTITUTE

We at our institute have a treatment plan based on current literature and our own experience.

- Counseling forms an integral part of the pretreatment planning. The patient and his family are made fully aware of what is involved in the treatment plan and the pros and cons of various options available. Patients are asked to choose the treatment option and even the chemotherapy regime where cost consideration is of paramount importance.
- T3-T4a N0 patients go for radical cystectomy first followed by adjuvant chemotherapy. T3-T4a patients with N1-3 disease in whom we think complete clearance is possible on preoperative evaluation such as imaging, EUA etc, we offer radical cystectomy followed by adjuvant chemotherapy. We believe that this gives a good p-stage and we rarely had to delay the chemotherapy by choosing surgery first. Adjacent visceral involvement such as uterus or vagina where anterior exenteration is possible, surgery first is practiced.
- Large bulky disease with non-gynecological adjacent visceral involvement such as rectum, large nodes that are unresectable with possible vascular/nerve injury, large nodal disease above inferior mesenteric vessels, metastasis above the pelvis, on preoperative evaluation, are candidates for neoadjuvant chemotherapy followed by surgery.
- M-VAC is the commonly used regime, four cycles for the adjuvant settings and three cycles in the neoadjuvant settings with a standard protocol followed for care during/after chemotherapy. If the cost is not an issue we offer them GC regime.
- Patients with S creatinine of >2.5 mg% are not offered chemotherapy, so also to the patients who have poor performance state and poor ejection fraction. Age is no bar provided their KS is satisfactory and patient has approximately two years of life expectancy.
- Chemotherapy is started at around six to eight weeks after the surgery. In case of neoadjuvant chemotherapy surgery is planned three weeks after the last cycle.
- Majority of the patients would have ileal conduit or Mainz II pouch rather than a neobladder as a policy.

## WHAT DO WE NEED TO DO IN INDIA?

Though this article is not aimed at looking at the ground realities in our country in the management of bladder cancer, we will all agree that bladder cancer patients do not reach the specialty centers where the urological oncological work is undertaken with the help of a medical and radiation oncology team and support team. The reason for having suboptimal treatments and results and at times losing these patients lies in the fact that this is not the job of occasional performers. We need to identify the places/institutions where this kind of work is done and this in turn would help them to achieve better results. We think this is the way forward.

In conclusion, there is a place for radical cystectomy in locally advanced T3b-T4 and N1-3 bladder cancer. Radical cystectomy alone rarely cures this subgroup of patients. There is increasing evidence that meticulous surgical clearance and extended lymphadenectomy has significant impact on disease-free survival Adjuvant chemotherapy has been found to be effective in terms of recurrence-free survival and better than cystectomy alone. Neoadjuvant chemotherapy followed by radical cystectomy also has beneficial effects in terms of downstaging the disease and improving recurrence-free survival. This perioperative chemotherapy (adjuvant/neoadjuvant) has 5-7% survival benefit and 10% reduction in death due to cancer disease. Excellent five-year survival rates have been achieved in patients achieving pT0 stage at surgery following chemotherapy (around 80%) and overall 40% five-year survival in node positive patients, which is promising. Though practiced widely, perioperative chemotherapy is not considered as a standard of care as yet. Current ongoing trials are likely to help us reaching the consensus over this. There is no role of preoperative or postoperative radiotherapy in locally advanced/metastatic bladder cancer except in non-TCC bilharzial/squamous cell carcinoma of bladder. Use of nomograms and prognostic factor evaluation may help us in the future in predicting disease relapse and may help us in tailoring the treatment accordingly. Newer and more effective chemotherapeutic drugs and ongoing trials will have significant impact on the treatment strategies and outcome of these patients in future.

## References

[1] Parkin DM, Whelan SL, Ferlay J (2002). Cancer incidence in five continents.

[2] Tongaonkar H, Shrivastava (2005). Bladder carcinoma. Evidence Based Management Guidelines Head -Neck, Cervical and Urological Cancers.

[3] Scher H, Bahnson R, Cohen S, Eisenberger M, Herr H, Kozlowski J (1998). NCCN urothelial cancer practice guidelines: National Comprehensive Cancer Network. Oncology (Williston Park).

[4] Oosterlinck W, Lobel B, Jakse G, Malmström PU, Stöckle M, Sternberg C (2002). Guidelines on bladder cancer. Eur Urol.

[5] Schrag D, Mitra N, Xu F, Rabbani F, Bach PB, Herr H (2005). Cystectomy for muscle-invasive bladder cancer: Patterns and outcomes of care in the Medicare population. Urology.

[6] Dalbagni G, Genega E, Hashibe M, Zhang ZF, Russo P, Herr H (2001). Cystectomy for bladder cancer: A contemporary series. J Urol.

[7] Stein JP, Lieskovsky G, Cote R, Groshen S, Feng AC, Boyd S (2001). Radical cystectomy in the treatment of invasive bladder cancer: Long term results in 1,054 patients. J Clin Oncol.

[8] Maderbacher S, Hochreiter W, Burkhard F, Thalmann GN, Danuser H, Markwalder R (2003). Radical cystectomy for bladder cancer today: Homogenous series without neoadjuvant therapy. J Clin Oncol.

[9] Herr HW (2003). Superiority of ratio based lymph node staging for bladder cancer. J Urol.

[10] Frank I, Cheville JC, Blute ML, Lohse CM, Nehra A, Weaver AL (2003). Transitional cell carcinoma of urinary bladder with regional node involvement treated by cystectomy: Clinicopathologic features associated with outcome. Cancer.

[11] Mills RD, Turner WH, Feishmann A, Markwalder R, Thalmann GN, Studer UE (2001). Pelvic metastasis from bladder cancer: Outcome in 83 patients after radical cystectomy and pelvic lymphadenectomy. J Urol.

[12] Soulie M, Straub M, Game X, Seguin P, De Petriconi R, Plante P (2002). A multicenter study of the morbidity of radical cystectomy in select elderly patients with bladder cancer. J Urol.

[13] Herr H, Lee C, Chang S, Lerner S, Bladder Cancer Collaborative Group (2004). Standardization of radical cystectomy and pelvic node dissection for bladder cancer: A collaborative group report. J Urol.

[14] Konety BR, Joslyn SA, O'Donnel MA (2003). Extent of pelvic lymphadenectomy and its impact on outcome in patients diagnosed with bladder cancer: Analysis of data from the surveillance, epidemiology and end results program database. J Urol.

[15] Paulsen AL, Hom T, Steven K (1998). Radical cystectomy: Extending the limits of pelvic node dissection improves survival for patients with bladder cancer confined to the bladder wall. J Urol.

[16] Quek ML, Stein JP, Clark PE, Daneshmand S, Miranda G, Cai J (2003). Natural history of surgically treated bladder carcinoma with extravesical tumour extension. Cancer.

[17] Herr HW, Donat SM (2001). Outcome of patients with grossly node positive bladder cancer after pelvic lymph node dissection and radical cystectomy. J Urol.

[18] Vieweg J, Gschwend JE, Herr HW, Fair WR (1999). The impact of primary stage on survival in patients with lymph node positive bladder cancer. J Urol.

[19] Logothetis CJ, Johnson DE, Chong C, Dexeus FH, Sella A, Ogden S (1988). Adjuvant cyclophosphamide, doxorubicin and cisplatin chemotherapy for bladder cancer: An update. J Clin Oncol.

[20] Skinner DG, Daniels JR, Lieskovsky G (1984). Current status of adjuvant chemotherapy after radical cystectomy for deeply invasive bladder cancer. Urology.

[21] Studer UE, Bacchi M, Biedermann C, Jaeger P, Kraft R, Mazzucchelli L (1994). Adjuvant cisplatin chemotherapy following cystectomy for bladder cancer: Results of a prospective randomized trial. J Urol.

[22] Pianezza O, Meneguelo M, Merlo F (2003). Adjuvant chemotherapy in locally advanced bladder cancer: Long term follow up of a multicenter study. J Urol.

[23] Otto T, Borgermann C, Krege S, Rubben H (2001). Adjuvant chemotherapy in locally advanced bladder cancer (pT3/pT4a, pN1-2, M0)-a phase III study. Eur Urol.

[24] Loehrer PJ, Einhorn LH, Elson PJ, Crawford ED, Kuebler P, Tannock I (1992). A randomized comparison of cisplatin alone or in combination with methotrexate, vinblastin and doxorubicin in patients with metastatic urothelial carcinoma: A comparative group study. J Clin Oncol.

[25] Stockle M, Meyenburg W, Wellek S, Voges G, Gertenbach U, Thüroff JW (1992). Advanced bladder cancer (stages pT3b, pT4a, pN1 and pN2): Improved survival after radical cystectomy and 3 adjuvant cycles of chemotherapy: Results of a controlled prospective study. J Urol.

[26] Stockle M, Meyenburg W, Wellek S, Voges GE, Rossmann M, Gertenbach U (1995). Adjuvant polychemotherapy of non -organ confined bladder cancer after radical cystectomy revisited: Long term results of a controlled prospective study and further clinical experience. J Urol.

[27] Lehmann J, Franzaring L, Thuroff J, Stockle M (2006). Complete long term survival data from a trial of adjuvant chemotherapy vs. control after radical cystectomy for locally advanced bladder cancer. BJU Int.

[28] Freiha F, Reese J, Torti FM (1996). A randomized trial of radical cystectomy versus radical cystectomy plus cisplatin, vinblastine and methotrexate chemotherapy for muscle invasive bladder cancer. J Urol.

[29] Sylvester R, Sternberg C (2000). The role of adjuvant combination chemotherapy after cystectomy in locally advanced bladder cancer: What we do not know and why. Ann Oncol.

[30] Advanced Bladder Cancer Meta-analysis Collaboration (2005). Adjuvant chemotherapy in invasive bladder cancer: A systemic review and meta-analysis of individual patient data. Eur Urol.

[31] Lehmann J, Retz M, Wiemers C, Beck J, Thüroff J, Weining C (2005). Adjuvant cisplatin plus methotrexate versus methotrexate, vinblastine, epirubicin and cisplatin in locally advanced bladder cancer: Results of a randomized, multicenter phase III trial (AUO trial AB5/95). J Clin Oncol.

[32] von der Maase H, Hansen SW, Roberts JT, Dogliotti L, Oliver T, Moore MJ (2000). Gemcitabine and cisplatin versus methotrexate, vinblastine, doxorubicin, and cisplatin in advanced or metastatic bladder cancer: results of a large, randomized, multinational, multicenter, phase III study. J Clin Oncol.

[33] Stroumbakis N, Herr HW, Cookson MS, Fair WR (1997). Radical cystectomy in octogenarian. J Urol.

[34] Bajorin DF, Dodd PM, Mazumdar M, Fazzari M, McCaffrey JA, Scher HI (1999). Long term survival in Metastatic Transitional cell carcinoma and prognostic factors predicting outcome of therapy. J Clin Oncol.

[35] (1999). Neoadjuvant cisplatin, methotrexate, and vinblastine chemotherapy for muscle-invasive bladder cancer: A randomized controlled trial. International collaboration of trialists. Lancet.

[36] Malmstrom PU, Rintala E, Wahlqvist R, Hellström P, Hellsten S, Hannisdal E (1996). Five year follow up of a prospective trial of radical cystectomy and neoadjuvant chemotherapy: Nordic cystectomy trial 1. J Urol.

[37] Sherif A, Holmberg L, Rintala E, Mestad O, Nilsson J, Nilsson S (2004). Neoadjuvant cisplatinum based combination chemotherapy in patients with invasive bladder cancer: A combined analysis of two Nordic studies. Eur Urol.

[38] Grossman HB, Natale RB, Tangen CM, Speights VO, Vogelzang NJ, Trump DL (2003). Neoadjuvant chemotherapy plus cystectomy compared with cystectomy alone for locally advanced bladder cancer. N Engl J Med.

[39] Millikan R, Dinney C, Swanson D, Sweeney P, Ro JY, Smith TL (2001). Integrated therapy for locally advanced bladder cancer: Final report of a randomized trial of cystectomy plus adjuvant MVAC versus cystectomy with both preoperative and post operative MVAC. J Clin Oncol.

[40] Winquist E, Kirchner TS, Segal R, Chin J, Lukka H, Genitourinary Cancer Disease Site Group, Cancer Care Ontario Program in Evidence-based Care Practice Guidelines Initiative (2004). Neoadjuvant chemotherapy for transitional carcinoma of the bladder: A systematic review and meta-analysis. J Urol.

[41] Logothetics CJ, Dexeus FH, Finn L, Sella A, Amato RJ, Ayala AG (1990). A prospective randomized trial comparing MVAC and CISCA chemotherapy for patients with metastatic urothelial tumours. J Clin Oncol.

[42] Logothetics C, Swanson D, Amato R, Banks M, Finn L, Ayala A (1996). Optimal delivery of perioperative chemotherapy: Preliminary results of a randomized, prospective, comparative trial of preoperative and post operative chemotherapy for invasive bladder carcinoma. J Urol.

[43] Herr HW, Donat SM, Bajorin DF (2001). Postchemotherapy surgery in patients with unresectable or regionally metastatic bladder cancer. J Urol.

[44] Sweeney P, Millikan R, Donat M, Wood CG, Radtke AS, Pettaway CA (2003). Is there a therapeutic role for post chemotherapy retroperitoneal lymph node dissection in metastatic transitional cell bladder cancer?. J Urol.

[45] Otto T, Krege S, Suhr J, Rübben H (2001). Impact of surgical resection of bladder cancer metastases refractory to systemic chemotherapy on performance score: A phase II trial. Urology.

[46] Smith JA, Crawford ED, Paradelo JC, Blumenstein B, Herschman BR, Grossman HB (1997). Treatment of advanced bladder cancer with combined preoperative irradiation and radical cystectomy versus radical cystectomy alone: A phase III intergroup study. J Urol.

[47] Huncharek M, Muscat J, Geschwind JF (1998). Planned preoperative radiation therapy in muscle invasive bladder cancer: Results of meta-analysis. Anticancer Res.

[48] Swanson DA, Liles A, Zagars GK (1990). Preoperative irradiation and radical cystectomy for stage T2 and T3 squamous cell carcinoma of the bladder. J Urol.

[49] Reisinger SA, Mohiuddin M, Mulholland SG (1992). Combined pre and post operative adjuvant radiation therapy for bladder cancer: A ten-year experience. Int J Radiat Oncol Biol Phys.

[50] Sarkis AB, Bajorin DF, Reuter VE, Herr HW, Netto G, Zhang ZF (1995). Prognostic value of p53 nuclear over expression in patients with invasive bladder cancer treated with neoadjuvant MVAC. J Clin Oncol.

[51] Bochner BH, Kattan MW, Vora KC, International Bladder Cancer Nomogram Consortium (2006). Postoperative nomogram predicting risk of recurrence following radical cystectomy for bladder cancer. J Clin Oncol.

[52] Karakiewicz PI, Shariat SF, Palapattu GS, Gilad AE, Lotan Y, Rogers CG (2006). Nomograms for predicting disease recurrence after radical cystectomy for transitional cell carcinoma of the bladder. J Urol.

[53] Bochner BH, Kattan MW, Vora KL (2007). Postoperative nomograms predicting risk of recurrence after radical cystectomy for bladder cancer. International Bladder Cancer Consortium. Urol Oncol.

[54] Karakiewicz PI, Shariat SF, Palapattu GS, Perrotte P, Lotan Y, Rogers CG (2006). Precystectomy nomogram for prediction of advanced bladder cancer stage. Eur Urol.

[55] Blackard CE, Byar DP, VACUR Group (1972). Results of a clinical trial of surgery and radiation in stage II and III carcinoma bladder. J Urol.

[56] Slack NH, Bross IDJ, Prout GR (1977). Five -year follow-up results of a collaborative study of therapies for carcinoma of the bladder. J Surg Oncol.

[57] Anderstrom C, Johansson S, Nilsson S, Unsgaard B, Wahlqvist L (1983). A prospective randomized study of preoperative radiation with cystectomy or cystectomy alone for invasive bladder carcinoma. Eur Urol.

[58] Awwad H, El-Baki HA, El-Bolkainy N (1979). Pre-operative irradiation of T3-carcinoma in bilharzial bladder: A comparison between hyperfractionation and conventional fractionation. Int J Radiat Oncol Biol Phy.

[59] Ghoneim MA, Ashmallah AK, Awwad HK, Whitmore WF (1985). Randomized trial of cystectomy with or without preoperative radiotherapy for carcinoma of the bilharzial bladder. J Urol.

